# Medical errors in Brazil

**Published:** 2019-09-10

**Authors:** Joao Furtado

**Affiliations:** 1 Department of Ophthalmology, University of São Paulo, Brazil

When there is an error, we are required to tell patients the truth, in a language they will understand. We also have to provide eye care free of charge for the related problems.

Everything about the patient's treatment (steps, procedures and medication) is recorded in the medical notes. If there is an error, the head of surgical care and the chief nurse would be notified, and an internal investigation would take place. This investigation would determine suggestions to prevent it from happening again (e.g., training or the development of a checklist).

